# Comprehensive histological investigation of age‐related changes in dermal extracellular matrix and muscle fibers in the upper lip vermilion

**DOI:** 10.1111/ics.12622

**Published:** 2020-06-11

**Authors:** T. Gomi, T. Imamura

**Affiliations:** ^1^ Cell Regulation Laboratory Bionics Program Tokyo University of Technology Graduate School of Bionics, Computer and Media Science 1404‐1 Katakura Hachioji Tokyo 192‐0982 Japan; ^2^ Frontier Research Center POLA Chemical Industries Inc 560 Kashio‐cho, Totsuka‐ku Yokohama Kanagawa 244‐0812 Japan

**Keywords:** ageing, lip, skin physiology, structure, statistics, vermilion

## Abstract

**Objective:**

Few histological studies have directly examined age‐related changes within the lips, although non‐invasive investigations of such changes are increasing. Therefore, this study aimed to provide histological and molecular data on age‐dependent alterations in the vermilion.

**Methods:**

Upper vermilion specimens from 15 female Caucasian cadavers (age range, 27–78 years) were investigated histologically or immunohistochemically.

**Results:**

Histologically, age‐dependent decreases in areas occupied by hyaluronan and collagenous fibres in the dermis of upper vermilion were demonstrated. Elastic fibre content varied widely between individuals. The area occupied by muscle fibres in the orbicularis oris muscle region within the vermilion also correlated negatively with age. Immunohistochemically, signals of four proteins were attenuated in vermilion from older individuals compared with young individuals: procollagen type I, hyaluronan synthase (HAS)1, myosin heavy chain (MYH)2 (a component of fast‐twitch oxidative muscle fibres) and MYH7 (a component of slow‐twitch muscle fibres). In contrast, signals of cell migration inducing hyaluronidase 1 (CEMIP) were intensified in vermilion from older individuals. No marked differences between young and older individuals were seen in procollagen type III, HAS2, HAS3, hyaluronidase (HYAL)1, HYAL2, MYH1 or MYH4.

**Conclusion:**

Age‐dependent decreases of hyaluronan in the dermis of vermilion were prominent, possibly due to both the decrease in synthesis (HAS1) and the increase in degradation (CEMIP). Furthermore, age‐dependent decreases in collagenous fibres and two types of muscle fibre in the vermilion were also identified histologically. Type I collagen, MYH2 and MYH7 appear to represent the molecules responsible for these respective decrements.

## Graphical abstract

The lips have been recognized the importance to facial attractiveness. Although various non‐invasive studies have demonstrated age‐dependent changes in the lip, only a few histological studies have directly examined age‐related changes within the lips. This article histologically demonstrates the age‐dependent changes of collagen and hyaluronan in the lips dermis, and MYH2 in the lip muscle for the first time.

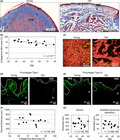

## Résumé

**Objectif:**

Peu d'études histologiques ont examiné directement les changements liés à l'âge sur les lèvres, bien que les enquêtes non invasives de ces changements soient en augmentation. Par conséquent, cette étude visait à fournir des données histologiques et moléculaires sur les altérations liées à l'âge dans le vermillon.

**Méthodes:**

Des échantillons de vermillon supérieur provenant de 15 cadavres de femme Caucasiens (tranche d'âge, 27‐78 ans) ont été étudiés histologiquement ou immuno‐histochimiquement.

**Résultats:**

Histologiquement, des diminutions dépendant de l'âge dans les zones occupées par l'hyaluronane et les fibres de collagène dans le derme du vermillon supérieur ont été démontrées. La teneur en fibres élastiques variait considérablement entre les individus. La zone occupée par les fibres musculaires dans la région du muscle orbiculaire oris au sein du vermillon était également corrélée négativement avec l'âge. Immuno‐histochimiquement, les signaux de quatre protéines ont été atténués dans vermillon des individus plus âgés que les jeunes: le procollagène type I, l'hyaluronane synthase (HAS) 1, la chaîne lourde de la myosine (MYH) 2 (un composant des fibres musculaires oxydatives à contraction rapide) et MYH7 (un composant des fibres musculaires à contraction lente). En revanche, les signaux du "cell migration inducing hyaluronidase 1 (CEMIP)" ont été intensifiés dans le vermillon des individus plus âgés. Aucune différence marquée entre les individus jeunes et âgés n'a été observée dans le procollagène type III, HAS2, HAS3, hyaluronidase (HYAL) 1, HYAL2, MYH1 et MYH4.

**Conclusion:**

Les diminutions dépendantes de l'âge du hyaluronane dans le derme du vermillon étaient importantes, probablement en raison à la fois de la diminution de la synthèse (HAS1) et de l'augmentation de la dégradation (CEMIP). En outre, les diminutions dépendantes de l'âge des fibres de collagène et de deux les types de fibres musculaires dans le vermillon ont également été identifiés histologiquement. Le collagène de type I, MYH2 et MYH7 semblent respectivement représenter les molécules responsables de ces diminutions.

## Introduction

The vermilion, the red part of the labium oris between the skin and oral mucosa generally called the lip by lay people, is the focus of much speculation regarding health conditions and influences on aesthetic appearance, due to its conspicuous central position in the lower face from the frontal view. For this reason, the vermilion is important to quality of life and has long attracted the attention of plastic surgeons and aesthetic and cosmetic researchers. A growing body of literature has recognized the importance of the vermilion to facial attractiveness in terms of factors such as height [1, 2] and the ratio of upper to lower vermilion heights [3]. Moreover, various non‐invasive studies have demonstrated age‐dependent changes in the vermilion, such as decreases in height and volume [4, 5, 6, 7, 8, 9, 10], softening [11, 12] and decreases in brightness and redness [12]. The vermilion is thus an important component of facial appearance that contributes to face‐to‐face communication, but changes with age. Aesthetic countermeasures to address decreases in vermilion volume, such as injection of autologous fat or hyaluronan, are currently common procedures for facial rejuvenation [13].

An increasing number of reports have described morphology‐oriented investigations and case reports of plastic surgery related to the vermilion, but there remains a paucity of histological studies directly examining alterations inside the vermilion. This is because the human vermilion is unique among primates, and no prominent vermilion is presented in any other animal species. Histological reports on the vermilion are available regarding the epithelium, muscle and blood vessels [14, 15, 16, 17]. Expressions of cytokeratin species in the stratified squamous epithelium covering the vermilion differ from those of the epidermis or oral mucosa [14]. Keratinization in the stratified squamous epithelium of the vermilion likewise differs from that of the epidermis overlying the skin [15]. Penna et al. have reported age‐related changes to the entire lip: the angle of the tip of the orbicularis oris muscle (OOM) in the lip curves superiorly in young individuals and becomes more linear with age; the OOM shows atrophy in older individuals; and thickness ratios of skin and hypodermal fat to total lip thickness in the middle of the upper white lip differ with age [16]. Age‐dependent decreases in blood vessels in the vermilion and a flattened dermo‐epithelial junction (DEJ) have recently been reported [17]. Some age‐related histological alterations to the vermilion are thus known, but have yet not fully clarified due to the lack of investigations concerning the dermis.

We therefore histologically investigated the dermis of upper lip vermilion to provide new data and a basis for a better understanding of lip ageing. Among the components of the dermis, we focused on the three principal dermal components of collagenous fibres, elastic fibres and hyaluronan. Collagen fibre bundles are a principal structure in the maintenance of three‐dimensional conformation and stiffness. Elastic fibres are also abundant in the dermis and are mechanically important due to their recoil ability [18]. Hyaluronan is the most abundant glycosaminoglycan in the interstitial space of the dermis and potentially influences both volume and viscosity in the dermis via the characteristics of binding large volumes of water and showing high viscosity [19].

In addition to the dermis, we investigated muscle fibres in the rounded knob‐like tip of OOM intruding into the vermilion. OOM comprises a considerable volume in the lip and extends into the vermilion, because the vermilion edge of the OOM curves towards the external side near the vermilion to form a J shape [16]. As noted above, atrophy of the OOM has been reported in older individuals [16], but the mechanisms underlying this atrophy have yet to be clarified. OOM is a skeletal muscle and these muscles mainly comprise four types of muscle fibre: fast‐twitch glycolytic fibres; fast‐twitch oxidative glycolytic fibres; fast‐twitch oxidative fibres and slow‐twitch fibres. Each of these is characterized by specific myosin heavy chain (MYH) proteins, named MYH1, MYH2, MYH4 and MYH7, respectively [20]. The present study therefore focused on these four MYH proteins.

## Materials and methods

### Tissue samples

**Figure 1:**
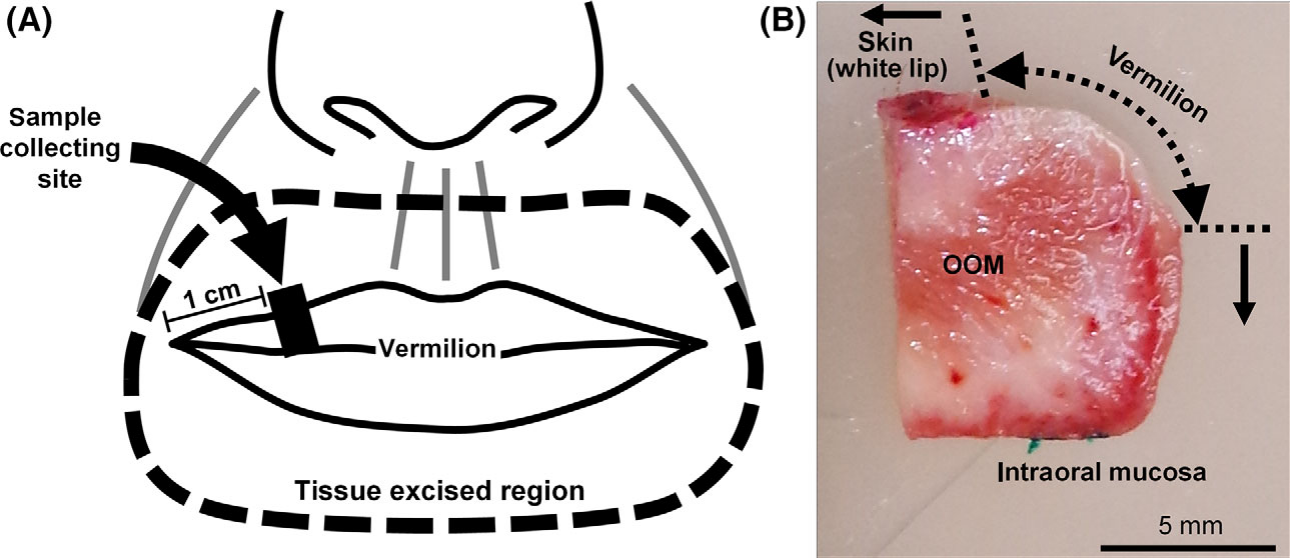
(A) Diagram of the site of sample collection in the tissue excised region: dotted line indicates the region of the tissue excised. Black square indicates the site of sample specimen collection for the analysis. (B) Representative image of a dissected specimen: the skin surface and intraoral labial mucosa on the oral cavity side were stained with red and green pigments, respectively. OOM, orbicularis oris muscle.

Human upper lip specimens from 15 female Caucasian cadavers (mean (±standard deviation) age 49.2 ± 15.8 years; range, 27–78 years) were provided by Obio, LLC (El Segundo, CA). The duration and storage condition from the death to the use in experiments are listed in Table S1. All specimens were identified using randomly assigned 6‐digit alphanumeric code during experimentation. The site of sample collection and dissected sample after cutting out were depicted in Fig. 1. The use of human tissue specimens was approved by the institutional review board of POLA Chemical Industries in 2017. The informed written consent of each donor for use of their body in research was confirmed by Obio, LCC.

### Histology

A 3‐mm width of upper lip vermilion was dissected from 1 cm medial to the right labial commissure and fixed in 10% neutral‐buffered formalin. Paraffin sections (thickness, 4 µm; vermilion surface‐to‐nose direction) were stained using Masson's trichrome (MT) and elastica van Gieson (EVG) and analysed using ImageJ software (National Institutes of Health, Maryland, USA). With MT staining, percentages of stained area (blue, collagenous fibres; red, muscle fibers) in each analysed area were quantified. The percentage of black‐stained area in each analysed area with EVG staining was quantified as a parameter for elastic fibre‐related extracellular matrix. Whole dermis was defined as the area between the DEJ and the superficial surface of the OOM, and upper dermis for analysis of EVG‐stained sections was defined as the dermis up to 100 µm below the DEJ. For detailed observations of collagenous fibres, fluorescence imaging of MT‐stained sections was performed with excitation wavelengths (Ex) of 615–645 nm and emission wavelengths (Em) of 655–760 nm for blue‐stained collagenous fibres, and Ex/Em of 530–560/570–650 nm for non‐collagenous protein/cells.

### Hyaluronan analysis

Eight‐micrometre thicknesses cryostat sections were fixed in the specific fixation solution (20 mL of formaldehyde solution, 140 mL of ethanol, 10 mL of acetic acid and 30 mL of phosphate‐buffered saline (PBS) [21]) at room temperature (RT) for 10 min. After blocking using an Avidin/Biotin Blocking Kit (Vector Laboratories, Burlingame, CA) and PBS containing 1% bovine serum albumin, sections were incubated overnight with 1:250 diluted biotinylated hyaluronan‐binding protein (HABP) (Hokudo, Sapporo, Japan) at 4°C, followed by visualization with fluorescein isothiocyanate‐conjugated streptavidin (Vector Laboratories). To confirm the binding specificity of HABP, a specimen digested by hyaluronidase from a *Streptomyces hyalurolyticus* (H1136; Sigma‐Aldrich, St. Louis, MO) was used as a control. Fluorescent area and intensity were quantitated using ImageJ software.

### Immunofluorescence

Rat monoclonal antibody to procollagen type I (clone M‐58, ab64409 at 1:100 dilution; Abcam, Cambridge UK), mouse monoclonal antibodies to HAS2 (clone 4E7, NBP2‐37446, 1:400; Novus Biologicals, Littleton, CO), MYH2 (clone TH81, NB100‐65675, 1:500; Novus Biologicals) and MYH4 (clone MF20, 53‐6503‐82, 1:1000; Thermo Fisher Scientific, Waltham, MA), rabbit polyclonal antibodies to procollagen type III (LS‐C664143‐200 1:400; LifeSpan BioSciences, Seattle, WA), HAS1 (HPA067602, 1:200; Atlas Antibodies, Stockholm, Sweden), HAS3 (LS‐B10150‐200, 1:100; LifeSpan BioSciences), cell migration inducing hyaluronidase 1 (CEMIP) (21129‐1‐AP, 1:25; Proteintech, Rosemont, IL), hyaluronidase (HYAL1) (ab85375, 1:100; Abcam), HYAL2 (PA5‐24223, 1:50; Thermo Fisher Scientific), MYH1 (bs‐5885R, 1:500; Bioss Antibodies, Woburn, MA) and MYH7 (22280‐1‐AP, 1:50; Proteintech) were used for primary antibodies. Isotype controls to each antibody were achieved by replacing the primary antibody with rat immunoglobulin (Ig)G (ab37361; Abcam), mouse IgG (ab18447; Abcam) or rabbit IgG (ab37361; Abcam), as respectively appropriate. Alexa Fluor 488 conjugated goat anti‐rat IgG (ab150165; Abcam), anti‐mouse IgG (ab150117; Abcam) and anti‐rabbit IgG (ab150081; Abcam) were used for secondary antibodies, as respectively appropriate. Cryostat sections (thickness, 6 µm) were fixed with 4% paraformaldehyde (Wako Pure Chemical Industries, Osaka, Japan) for 3 h at 4°C, followed by antigen retrieval performed using Target Retrieval Solution (Agilent, Santa Clara, CA) for 12 h at 60°C. After washing, sections were incubated with 0.1% Triton X‐100 for 10 min; this step was skipped in the case of HAS3, HYAL1 and HYAL2. After blocking in 10% normal goat serum (ab7481; Abcam) for 1 h at RT, sections were incubated with primary antibody for 12 h at 4°C. Sections were washed again, then incubated with secondary antibody for 1 h at RT.

### Statistics

The R‐package was used for all statistical analyses (The R Project, http://www.R‐project.org). Pearson's correlation coefficient was used for correlation analyses with values of *P* < 0.05 considered significant.

## Results

### Deteriorated collagenous fibres in the dermis of upper lip vermilion from older individuals

**Figure 2:**
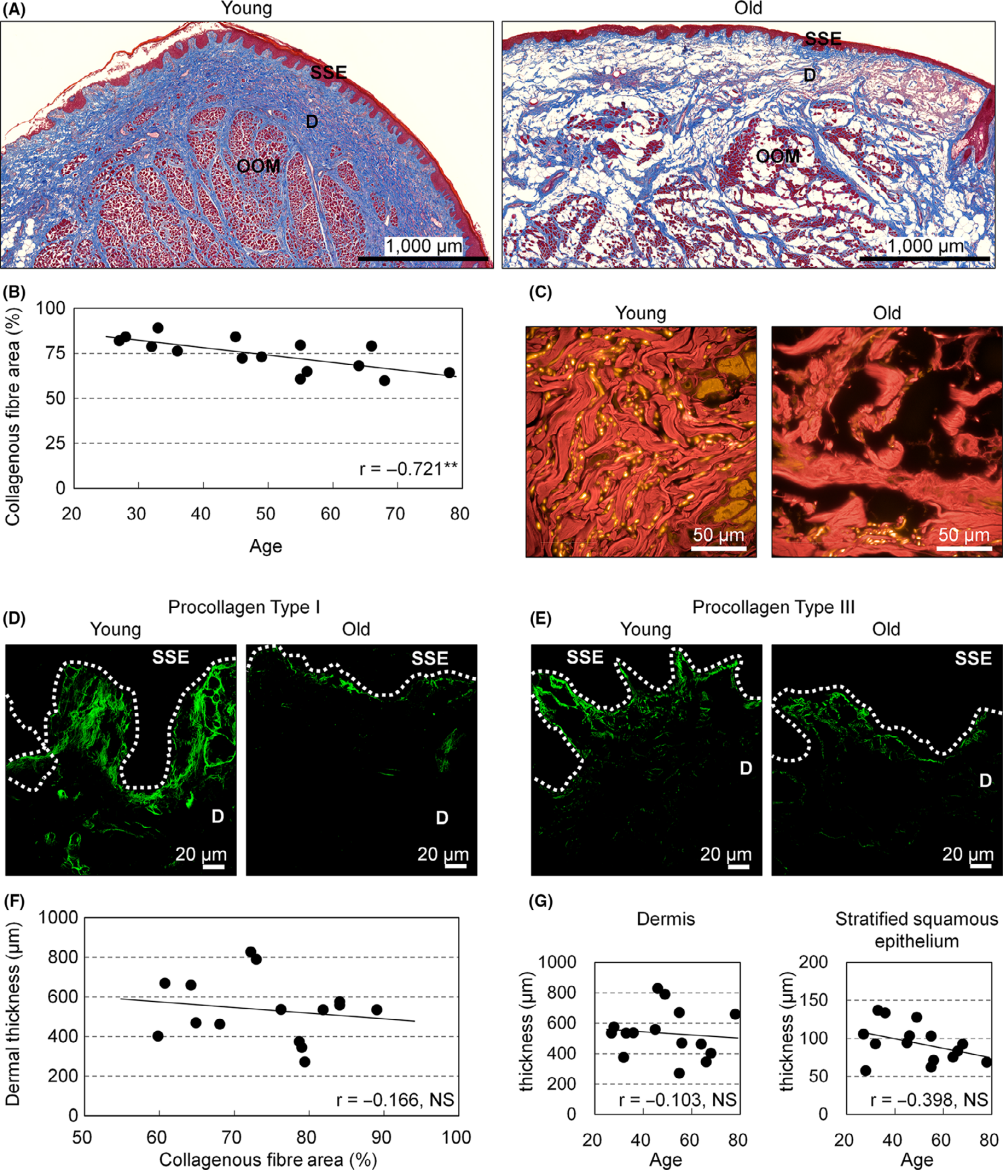
Changes in collagenous fibres and procollagens in the dermis of upper lip vermilion with age. (A) MT‐stained images of the upper lip vermilion at 27 (young) and 78 (old) years old. Bar = 1000 µm; blue, collagenous fibres; brown, other proteins (e.g. epithelial proteins or muscle proteins). SSE, stratified squamous epithelium; D, dermis; OOM, orbicularis oris muscle. (B) Changes in collagenous fibre area in the upper lip vermilion dermis with age (*n* = 15). **P < 0.01 (Pearson's correlation test). (C) High‐magnification fluorescence images of the mid‐dermis of the vermilion in MT‐stained section at 27 (young) and 78 (old) years old. Bar = 50 µm; red, collagenous fibres; yellow, other proteins. (D, E) Representative images of immunofluorescence for procollagen species at 27 (young) and 78 (old) years old. Green shows procollagen type I (D) and procollagen type III (E). Bar = 20 µm; dotted line, DEJ. (F) Thickness of the vermilion dermis was plotted against collagenous fibre area in the vermilion dermis (*n* = 15). NS, not significant (Pearson's correlation test). (G) Individual plots of the thickness of the dermis or the stratified squamous epithelium of upper lip vermilion according to age (*n* = 15). NS, not significant (Pearson's correlation test).

Collagenous fibres were observed sparsely in vermilion dermis obtained from older individuals, whereas dense collagenous fibres were apparent in vermilion obtained from young individuals (Fig. 2A). Collagenous fibre area in the vermilion dermis correlated negatively with age (*r* = −0.721, *P* = 0.003; Fig. 2B). Observation of fluorescence in MT‐stained sections at high magnification revealed dense, regularly arranged collagen fibre bundles in young individuals, but thin, poorly organized swollen collagen fibres in older individuals (Fig. 2C) in the mid‐dermis. To investigate whether synthesis of collagen fibre proteins was involved in these age‐dependent deteriorations in collagenous fibres, analyses for procollagen type I and type III were conducted. Immunofluorescence for procollagen type I was strong just beneath the stratified squamous epithelium in both young and older individuals. This immunolabelling was widespread throughout the papillary dermis and some signals were also detected in the mid‐dermis in young individuals, but was localized to just beneath the stratified squamous epithelium in older individuals (Fig. 2D). In contrast, procollagen type III showed no marked differences between specimens, although strong signals just beneath the stratified squamous epithelium were also shown in both young and older individuals (Fig. 2E). Collagenous fibres occupy most of the dermis in the vermilion and could be expected to have a pronounced influence on dermal volume. We therefore analysed the relationship between collagenous fibre area and dermal thickness in the vermilion dermis, but found no correlation between these parameters (*r* = −0.166, *P* = 0.553; Fig. 2F). Mean distance from DEJ to OOM, which representing dermal thickness, was 534.0 ± 156.8 µm, and mean distance from the surface of the stratified squamous epithelium to the DEJ, which indicating the thickness of the stratified squamous epithelium, was 93.8 ± 25.1 µm; neither of these values showed any correlation with age (Fig. 2G).

### High variation of elastic fibres in the dermis of upper lip vermilion between individuals

**Figure 3:**
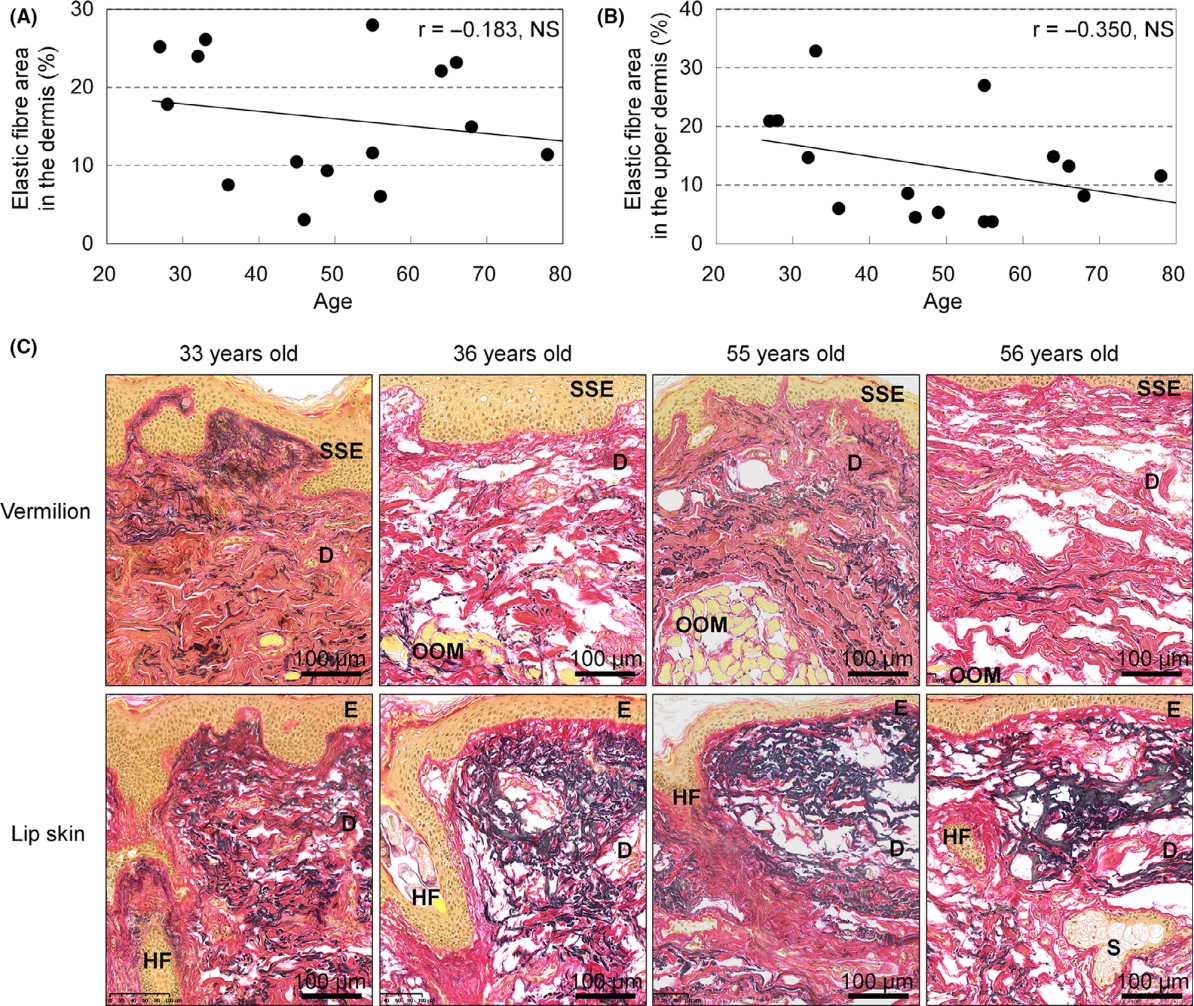
Elastic fibres in the dermis of upper lip vermilion at different ages. (A) Plots of elastic fibre area stained by EVG according to age (*n* = 15). NS, not significant (Pearson's correlation test). (B) Plots of elastic fibre area in the upper dermis (area between DEJ and 100 µm deep to DEJ) of the vermilion according to age (*n* = 15). NS, not significant (Pearson's correlation test). (C) Representative images of EVG‐stained upper lip vermilion and white lip skin adjacent to the border of the upper lip vermilion. Bar = 100 µm; black, elastic fibres; red, collagen or stratum corneum; yellow, other proteins (e.g. epithelial proteins or muscle proteins). SSE, stratified squamous epithelium; D, dermis; OOM, orbicularis oris muscle; E, epidermis; HF, hair follicle; S, sebaceous gland.

The black‐stained area representing elastic fibres with EVG staining in the vermilion dermis showed no correlation with age (*r* = −0.183, *P* = 0.513; Fig. 3A). We further analysed these elastic fibres by limiting the analysed area to a depth of 100 µm below the DEJ, because the terminal region of elastic fibres in the upper dermis is considered more sensitive to ageing [22]. However, results for elastic fibres in the upper dermis of vermilion still showed no correlation with age (*r* = −0.350, *P* = 0.200; Fig. 3B). Elastic fibres showed large individual variations even within the same generation, and this variation was observed even if similar levels of elastosis were seen in surrounding skin of the white lip (Fig. 3C).

### Age‐dependent decrease in hyaluronan in the dermis of upper lip vermilion

**Figure 4:**
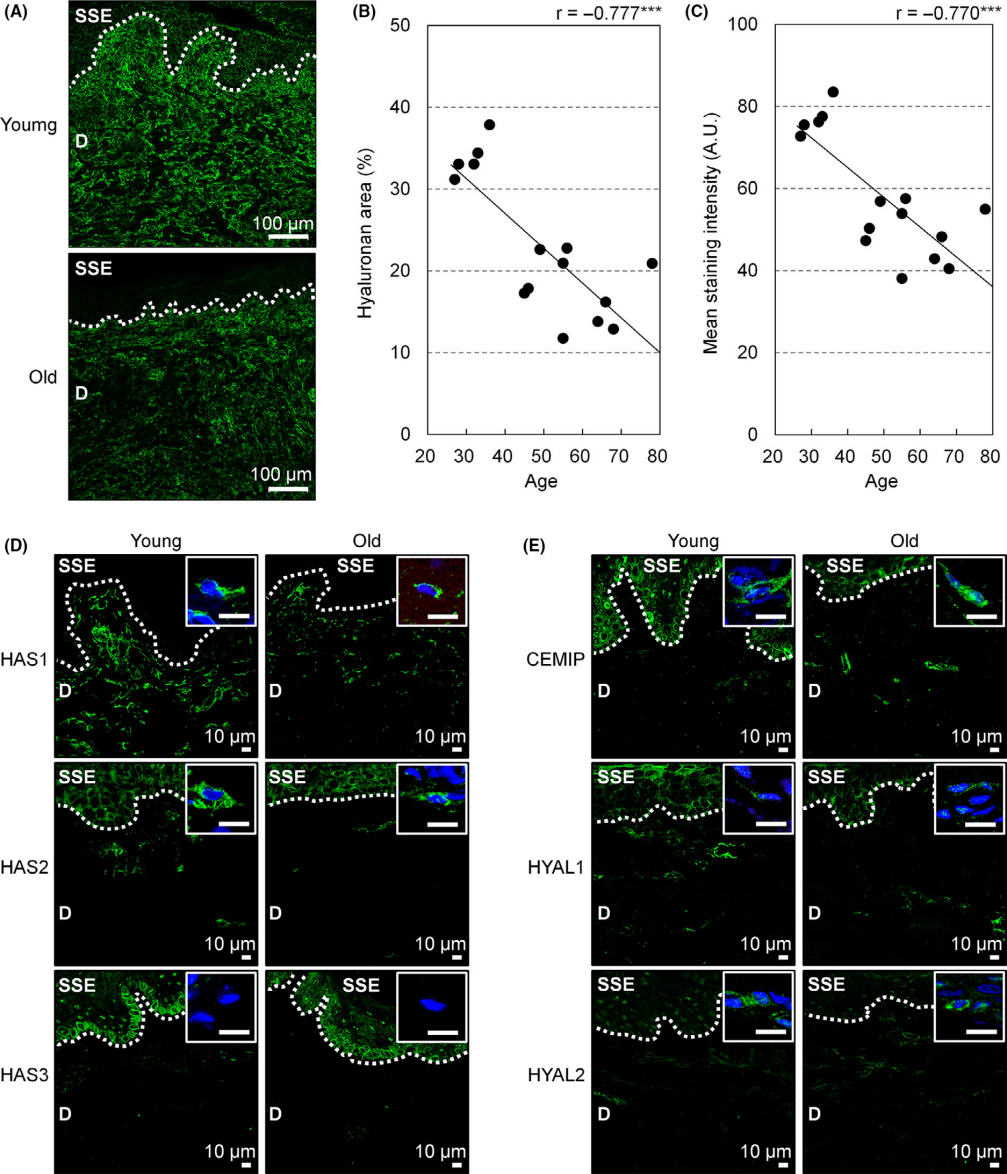
Negative correlation between hyaluronan in the dermis of upper lip vermilion and age, and immunofluorescence for enzymes involved in hyaluronan metabolism. (A) Representative images of hyaluronan staining. Bar = 100 µm; green, hyaluronan; dotted line, DEJ; SSE, stratified squamous epithelium; D, dermis. (B, C) Plots of stained area (B) and staining intensity (C) of fluorescence labelling for hyaluronan in the dermis of upper lip vermilion according to age. *n* = 15 each. ***P < 0.001 (Pearson's correlation test). (D, E) Representative images of immunofluorescence for hyaluronan‐related enzymes. Green indicates immunolabelled signal from antibody specifically against HAS1 (D, top), HAS2 (D, middle), HAS3 (D, bottom), CEMIP (E, top), HYAL1 (E, middle) and HYAL2 (E, bottom). Inserts represent high‐magnification images of dermal cells with DAPI. Donor age in each image was 27 years (young) and 78 years (old). Blue; nuclei; bar = 10 µm; dotted line, DEJ; SSE, stratified squamous epithelium; D, dermis.

The dermal area stained positively for HABP was greater in young individuals than in older individuals (Fig. 4A). Correlation analysis showed an age‐dependent decrease in the area staining for hyaluronan (*r* = −0.777, *P* < 0.001; Fig. 4B). The fluorescent intensity of HABP staining also correlated negatively with age in a similar fashion to that of the stained area (*r* = −0.770, *P* < 0.001; Fig. 4C). To explore the candidate mechanisms underlying this age‐dependent reduction in hyaluronan, we conducted immunofluorescence investigations for three hyaluronan synthases (HAS1, HAS2 and HAS3; Fig. 4D) and for three enzymes associated with hyaluronan degradation (CEMIP, HYAL1 and HYAL2; Fig. 4E). The results showed reduced immunofluorescence for HAS1 in the vermilion dermis from older individuals, but comparable levels of immunofluorescence for HAS2 between young and older individuals and detection of HAS3 only in the squamous epithelium, not in the dermis (Fig. 4D). Immunofluorescence for CEMIP was increased in the vermilion dermis from older individuals, whereas no marked differences in HYAL1 or HYAL2 were seen between young and older individuals (Fig. 4E).

### Decreased MYH2 and MYH7 in the OOM in vermilion from older individuals

**Figure 5:**
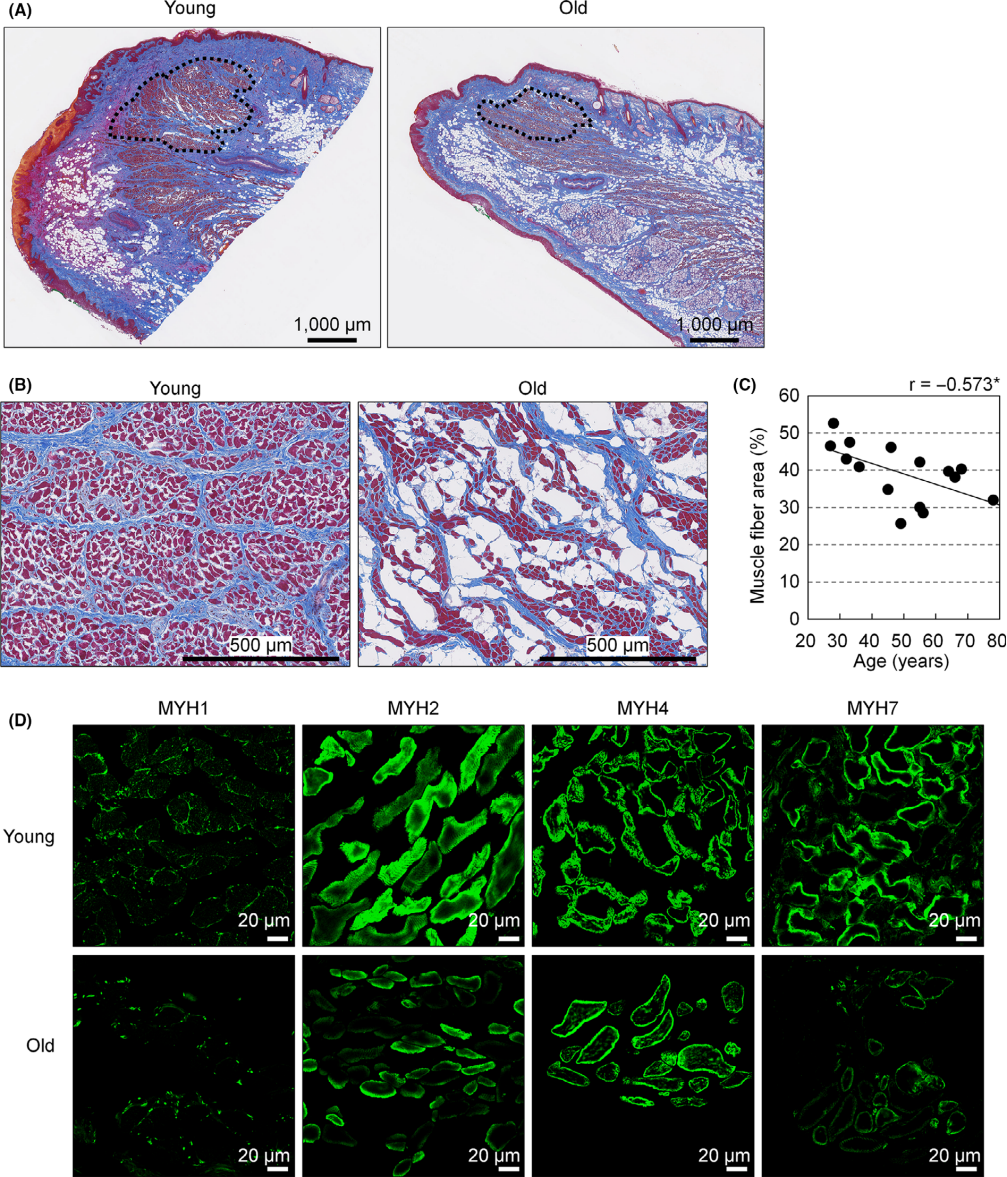
Muscle fibres and related myosin heavy chains in the upper lip vermilion at different ages. (A) Representative low‐magnification images of MT‐stained sections of the upper lip vermilion at 33 (young) and 66 (old) years old. Bar = 1000 µm; dotted line, analysed regions as the rounded knob‐like tip of OOM; red, muscle fibres; blue, collagenous fibres. (B) High‐magnification images of muscle fibre in the rounded knob‐like tip of the OOM in upper lip vermilion from 27 (young) and 78 (old) years old. Bar = 500 µm; red, muscle fibres; blue, collagenous fibres. (C) Plots of areas occupied by muscle fibres against age. *n *= 15. *P < 0.05 (Pearson's correlation test). (D) Representative images of immunofluorescence for myosin heavy chain proteins at 27 (young) and 78 (old) years old. Green indicates immunolabelled signal by antibodies specifically targeting MYH1, MYH2, MYH4 and MYH7, respectively. Bar = 20 µm.

Similar to a previous report [16], the tip of the OOM intruded into the vermilion and formed a rounded knob‐like shape in young individuals, but those features were reduced in older individuals (Fig. 5A), and muscle fibres in this region also displayed increasing atrophy with age (Fig. 5B). We therefore analysed the area occupied by muscle fibres in the rounded knob‐like tip of OOM. As expected, a negative correlation was seen between muscle fibre area and age (*r* = −0.573, *P* = 0.025; Fig. 5C). In comparisons of immunofluorescence results for specimens obtained from young and older individuals, MYH2 and MYH7 were decreased in older individuals, whereas no marked differences were seen for MYH4 (Fig. 5D). Levels of MYH1 were minimal even in specimens obtained from young individuals, making comparison between age groups difficult (Fig. 5D).

## Discussion

The present study clearly identified a prominent age‐dependent decrease in hyaluronan in the vermilion dermis. Although there are a few discrepancies in changes to hyaluronan with age in both intrinsically aged skin [23, 24, 25] and photodamaged skin [25, 26, 27, 28], similar age‐dependent decreases in hyaluronan have been reported in surrounding tissues such as the gingiva [29] and the skin [23, 24, 25, 26, 27, 28]. The present study also revealed decreased HAS1, an enzyme involved in hyaluronan synthesis, and increased CEMIP, an enzyme involved in hyaluronan degradation in the vermilion dermis of older individuals. Summarizing the hyaluronan‐related data presented in this study, hyaluronan decreases with age as a result of both decreased synthesis and increased degradation, and plausible candidates for responsible enzymes include HAS1 for synthesis and CEMIP for degradation.

The present study also showed decreases in the area occupied by collagenous fibres reflecting a decreasing abundance of collagen, and deteriorations in the morphology of collagen fibre bundles in the vermilion dermis from older individuals. Collagenous fibres in the vermilion dermis thus change with age in a similar fashion to those generally accepted in the dermis of the skin [18, 30]. More detailed analyses in further investigations those including degradative proteinases and molecules involved in collagen fibre formation, are required to shed light on the factors causing changes in collagenous fibres of the vermilion dermis. However, the present result of decreased procollagen type I levels in the vermilion dermis from older individuals suggests that attenuation of new collagen synthesis is probably be involved in the age‐dependent collagen decrease in the vermilion dermis.

Intrinsic ageing and photodamage are generally considered as major factors contributing to age‐dependent decreases in hyaluronan and collagen. The decrease in HAS1 is consistent with previously reported changes in both intrinsically aged and photoaged skin [26, 28, 31], and decreased levels of procollagen type I and consistently low levels of procollagen type III also agree with reported changes to each type of collagen in intrinsically aged skin [32]. The finding of increased CEMIP agrees well with results for photoaged skin [26], whereas no certain information appears to have been reported regarding CEMIP in intrinsically aged skin. The result in procollagen type I also showed good agreement with changes in procollagen type I in photodamaged skin [33]. By contrast, regarding HAS2 and HAS3, our results run counter to findings reported for the skin [28, 31]. However, the difference in results for HAS2 may be explained by differences in detection targets (i.e. mRNA vs. protein). The difference in results for HAS3 may be attributable to localized expression. Expression of HAS3 may be localized to the stratified squamous epithelium in the vermilion. With regard to the hyaluronidases evaluated in the present study, decreased HYAL2 has been reported in skin from aged individuals [31], but no certain information appears to have been reported regarding HYAL1. The results for procollagen type III also contradict findings in photodamaged skin [33]. The cause of this difference may involve the influences of mechanical stress and site‐specific characteristics of fibroblasts. Mechanical stress modulates expressions of procollagen type I and type III individually, and effects of mechanical stress differ among the tissues from which fibroblasts originate [34]. Vermilion moves larger distances more frequently than facial skin and would thus be likely to experience more mechanical stress in terms of both intensity and frequency. The application of mechanical stresses to intrinsically aged or photodamaged fibroblasts may cause different results in terms of procollagen type III synthesis compared to that from non‐stressed fibroblasts. From the perspective of photodamage, the high level of variation in elastic fibres shown in the present study was thought to reflect the degree of photo‐exposure of the vermilion. Little relationship between progression of elastosis, a hallmark of photoaged skin [35], in neighbouring skin and the condition of elastic fibres in the vermilion dermis was also observed. The lip protrudes compared to other parts of the face, so neighbouring labial skin on the upper vermilion border always faces upward and is frequently exposed to sunlight. By contrast, the angle of the vermilion generally varies between individuals. High levels of individual variation might thus be explained by differing degrees of exposure of the vermilion to sunlight due to differences in vermilion angle among individuals. In addition, elastic fibres show elastolysis with intrinsic ageing and elastosis with photoageing in the skin [35], and in gingiva, oxytalan fibres and elaunin fibres (two of the three fibre types in the elastic fibre system) decrease with intrinsic age, whereas elastic fibres do not [22]. Site‐specific characteristics thus could well be occurring in elastic fibre alterations. Site‐specific characteristics of the vermilion dermis adding to photo‐induced elastosis may thus lead to complicated results for elastic fibres in the vermilion dermis. Some issues thus remain a little controversial and necessitate further investigations such as donor background control, morphological measurements and histological analyses from fresh biopsy sample. However, our results of decreased HAS1 and procollagen type I and increased CEMIP suggest that the lip vermilion may be affected by sun exposure.

Collagenous fibres would be expected to influence dermal volume, because they maintain the three‐dimensional conformation of the dermis. In addition, collagenous fibres are also central to the mechanical properties of the dermis, because degradative morphological changes in collagenous fibres correlate with the viscoelasticity of the skin [36]. However, levels of collagen did not correlate with the dermal thickness of the vermilion in the present study. Moreover, the thickness of the vermilion dermis did not correlate with age in this study. Given previous non‐invasive measurements, the dermis instead could have been expected to thin with age [4, 5, 6, 7, 8, 9, 10]. This may be attributable to the wide interindividual variability, even within comparable age strata. Although further investigations of additional cases may help clarify this issue, a smaller contribution to the entire thickness of the upper vermilion by the dermis than the OOM is considered as another possibility, because morphological changes in the OOM to become more linear with age may flatten the curvature of the lip and reduce the visible vermilion [16]. In addition, the decreased thickness in previous non‐invasive investigations resulted from measurement of the entire lip thickness, including the OOM [7, 8, 9, 10]. Although the relationship between the amount of collagen and the thickness of the vermilion dermis thus cannot be discussed with any clarity using only the present results, our findings of decreased and deformed collagenous fibres should contribute to explanations for the softening of the vermilion in older individuals [11, 12].

In addition to dermal extracellular matrix, a decrease in muscle fibres within the OOM in the vermilion was confirmed in this study, supporting previous observations [16]. We also revealed that the portion of the OOM extending into the vermilion consisted mainly of MYH2, −4 and −7, indicating that OOM contains both fast‐ and slow‐twitch muscle fibres, again agreeing with previous reports [37, 38]. Decreases in MYH2 and MYH7 in older individuals resemble those seen for the soleus muscle [39] and suggest losses of MYH2 and MYH7 as causes of age‐dependent muscle fibre atrophy in the OOM. Although muscle fibres shift its type depending on muscle species and stimulations [39, 40, 41], decreased MYH2 and MYH7 in combination with a constant MYH4 level suggests oxidative‐ to glycolytic‐type changes in OOM with age, because MYH2, −4 and −7 are myosin heavy chains for fast‐twitch oxidative glycolytic muscle fibres, fast‐twitch glycolytic muscle fibres and slow‐twitch oxidative fibres, respectively.

The present study has some limitations accompanying cadaveric study. The first relates to the variation of the length of the period from death to providing specimens for investigation, because the timing of donor occurring is uncontrollable. The samples used in this investigation were collected after autopsy from donors whom died by accident or diseases. However, despite the possible variations in the sample freshness, we found that major outcomes in the present study, such as collagen, elastic fibre, hyaluronan, muscle fibres, were uncorrelated with storage periods (Table S2). Therefore, changes in collagen, hyaluronan and muscle fibres that have revealed in the present study are considered to have been caused by ageing. Second, the morphology of the lip tissue sometime varies between on the living human and post‐resection from the dead body. The morphometric assessment, therefore, were not possible in the present study. Also, additional deformation of tissues during freezing/dissection might have occurred, because the specimens were isolated from the deceased and then frozen. To eliminate these potential problems, we analysed the samples according to per unit area. Further limitation is a lack of the donors’ background information, such as smoking history and sun exposure frequency, during their lifetimes. Those habits should affect on tissue ageing. Collecting such information regarding the deceased donor who died in an accident or unpredictable diseases is highly challenging, unlike the samples donated by living individuals for research purposes. Future investigations using both morphological measurements and non‐invasive assessment of living human tissue would overcome these issues.

In conclusion, the present study appears to provide the first histological evidence of age‐dependent decreases to collagen and hyaluronan in the dermis of the vermilion, and reductions to MYH2 and MYH7 in the aged OOM in the vermilion. These lines of evidence provide a basis for age‐related research in the lips.

## Conflict of interests

T.G. is an employee of POLA Chemical Industries. T.I. declares that he has no conflicts of interest.

## Supporting information


**Table S1.** Storage conditions and lengths of time between each major experimental point, and freezing methods.

**Table S2.** Results of correlation analysis between the length of storage time of each storage conditions and major outcome.
