# Supplementary material for: Comprehensive histological investigation of age‐related changes in dermal extracellular matrix and muscle fibers in the upper lip vermilion
Source: Int J Cosmet Sci. 2020 Jun 11;42(4):359–68. doi: 10.1111/ics.12622 (PMC7496161; doi:10.1111/ics.12622)
Supplement: Supplementary file 1 — Table S1. Storage conditions and lengths of time between each major experimental point, and freezing methods. [file ICS-42-359-s001.docx]

**Table S1. Storage conditions and lengths of time between each major experimental point, and freezing methods.**

| Donor  age | Time between  death to sampling^1^ (hours, at 4°C) | Freezing method | Time from freezing to  dissection for histology^2^ (hours, at -80°C) |
| --- | --- | --- | --- |
| 27 | 277 | Acetone/dry ice bath | 1320 |
| 28 | 247 | Liquid nitrogen | 2976 |
| 32 | 204 | Acetone/dry ice bath | 4824 |
| 33 | 94 | Liquid nitrogen | 3480 |
| 36 | 137 | Liquid nitrogen | 2952 |
| 45 | 253 | Acetone/dry ice bath | 3504 |
| 46 | 217 | Acetone/dry ice bath | 5328 |
| 49 | 199 | Liquid nitrogen | 1320 |
| 55 | 110 | Liquid nitrogen | 2256 |
| 55 | 82 | Liquid nitrogen | 2280 |
| 56 | 290 | Acetone/dry ice bath | 480 |
| 64 | 88 | Liquid nitrogen | 4176 |
| 66 | 183 | Acetone/dry ice bath | 4824 |
| 68 | 249 | Acetone/dry ice bath | 4152 |
| 78 | 110 | Liquid nitrogen | 7056 |

^1^ Lip specimens were excised after an examination such as inquest and autopsy.

^2^ Medical deep freezers were used for storage, except during transportation. Dry ice was used during transportation.
