# Supplementary material for: Comprehensive histological investigation of age‐related changes in dermal extracellular matrix and muscle fibers in the upper lip vermilion
Source: Int J Cosmet Sci. 2020 Jun 11;42(4):359–68. doi: 10.1111/ics.12622 (PMC7496161; doi:10.1111/ics.12622)
Supplement: Supplementary file 2 — Table S2. Results of correlation analysis between the length of storage time of each storage conditions and major outcome. [file ICS-42-359-s002.docx]

**Table S2. Results of correlation analysis between the length of storage time of each storage conditions and major outcome**

| Percentage of area  in region of interest | Time between  death to sampling^1^ (hours, at 4°C) | Time from freezing to  resection for histology^2^ (hours, at -80°C) |
| --- | --- | --- |
| Collagenous fiber area  in the dermis (%) | r = 0.049  p = 0.862 | r = -0.099  p = 0.725 |
| Elastic fiber area  in the dermis (%) | r = -0.304  p = 0.271 | r = 0.012  p = 0.967 |
| Hyaluronan stained area  in the dermis (%) | r = 0.079  p = 0.778 | r = -0.184  p = 0.512 |
| Muscle fiber area  in the OOM (%) | r = 0.039  p = 0.890 | r = 0.196  p = 0.483 |

r, Pearson's correlation coefficient; p, p-value of Pearson's correlation test.

^1^ Lip specimens were excised after an examination such as inquest and autopsy.

^2^ Medical deep freezers were used for storage, except during transportation, and dry ice was used during transportation.
